# Examining Brain Function Changes in HIV‐Infected Patients With Asymptomatic Neurocognitive Impairment: A Longitudinal Study

**DOI:** 10.1002/brb3.70559

**Published:** 2025-06-26

**Authors:** Juming Ma, Zhongkai Zhou, Shuai Han, Chuanke Hou, Xingyuan Jiang, Fan Xu, Haixia Luo, Jiaojiao Liu, Wei Wang, Haiyan Zhao, Lingling Zhao, Hongjun Li

**Affiliations:** ^1^ Department of Radiology Qilu Hospital of Shandong University Jinan China; ^2^ Department of Radiology Beijing Youan Hospital Capital Medical University Beijing China; ^3^ The Sixth People's Hospital of Zhengzhou Zhengzhou China; ^4^ Beijing Advanced Innovation Centre for Biomedical Engineering Beihang University Beijing China

**Keywords:** amplitude of low‐frequency fluctuation, asymptomatic neurocognitive impairment, functional connectivity, HIV‐associated neurocognitive disorders, regional homogeneity

## Abstract

**Background:**

HIV‐associated neurocognitive disorders (HAND), especially asymptomatic neurocognitive impairment (ANI), the initial stage of HAND, persist among a substantial proportion of individuals living with HIV despite the introduction of combination antiretroviral therapy (cART). Resting‐state functional magnetic resonance imaging (rs‐fMRI) focusing on ANI among HIV‐related patients is rarely reported.

**Methods:**

60 right‐handed Chinese male patients with HIV‐associated ANI underwent baseline and follow‐up neurocognitive examination, and rs‐fMRI scans over an average interval of 1.68 years. Brain function alterations were evaluated through amplitude of low‐frequency fluctuation (ALFF/fALFF), regional homogeneity (ReHo), and functional connectivity (FC) analyses.

**Results:**

In this study, significant reductions in ALFF were observed in the MOG, cuneus, superior frontal gyrus, and supplementary motor area in the follow‐up group compared to baseline. This was accompanied by increased ALFF in the right insula. ReHo analysis revealed decreased values in the left median cingulate, right calcarine fissure, MOG, and left precentral gyrus, alongside increased ReHo in the supramarginal gyrus, postcentral gyrus, parahippocampal gyrus, and calcarine fissure. FC analysis demonstrated decreased connectivity between the precentral gyrus and calcarine cortex and between the MOG and calcarine cortex. Correlation analysis indicated that these imaging changes were correlated with declines in specific neurocognitive domains, including memory, speed of information processing, and executive function.

**Conclusion:**

Our research demonstrates a gradual deterioration in brain function in HIV‐positive individuals with ANI despite receiving cART. This decline correlates with worsening neurocognitive abilities, specifically visual processing and executive function.

## Graphical abstract

This study investigated resting‐state functional magnetic resonance imaging (rs‐fMRI) focusing on asymptomatic neurocognitive impairment (ANI) among HIV related patients. The results found a gradual deterioration in brain function in HIV‐positive individuals with ANI despite receiving cART. This decline correlates with worsening neurocognitive abilities, specifically visual processing and executive function.

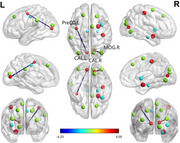

**Abbreviations**
ADL: Activities of Daily Living
ALFF: Amplitude of Low‐Frequency Fluctuation
AMYG: Amygdala
ANI: Asymptomatic Neurocognitive Impairment
BDI: Beck Depression Inventory
CAL: Calcarine Fissure and Surrounding Cortex
cART: Combination Antiretroviral Therapy
DCG: Median Cingulate and Paracingulate Gyri
fALFF: Fractional Amplitude of Low‐Frequency Fluctuation
FC: Functional Connectivity
FDR: False Discovery Rate
HAND: HIV‐associated Neurocognitive Disorders
INS: Insula
IQR: Interquartile Range
LING: Lingual Gyrus
MNI: Montreal Neurological Institute
MOG: Middle Occipital Gyrus
MRI: Magnetic Resonance Imaging
PHG: Parahippocampal Gyrus
PoCG: Postcentral Gyrus
PreCG: Precentral Gyrus
ReHo: Regional Homogeneity
ROI: Region of Interest
SD: Standard Deviation
SFGdor: Superior Frontal Gyrus, Dorsolateral
SMA: Supplementary Motor Area
SMG: Supramarginal Gyrus
SPSS: Statistical Package for the Social Sciences
TND: Target Not Detected

## 1 Introduction

During the initial phase of HIV infection, virus‐infected macrophages infiltrate the central nervous system, leading to enduring harm to nerve cells (Dash 2023). Despite combination antiretroviral therapy (cART), approximately half of individuals with HIV experience sensory, motor, or neurocognitive deficits, collectively known as HIV‐associated neurocognitive disorders (HAND) (McMahan et al. 2023). HAND manifests in three progressive stages: asymptomatic neurocognitive impairment (ANI), mild neurocognitive disorder (MND), and HIV‐associated dementia (HAD) (Hirsch et al. 2022). ANI, the earliest phase, is characterized by subtle or absent symptoms (Zenebe et al. 2022). Timely identification and intervention during this stage can potentially delay or prevent the onset of HAD, thereby enhancing patient outcomes.

Resting‐state functional magnetic resonance imaging (rs‐fMRI) is a non‐invasive method used to observe neuronal activity without ionizing radiation, achieved through the detection of the blood oxygenation level‐dependent (BOLD) signal (Marapin et al. 2023). In the brain, neurons receive oxygen through hemoglobin in capillaries. When neural activity increases, oxygen consumption rises, leading to enhanced local blood flow. Oxygenated hemoglobin is diamagnetic, while deoxygenated hemoglobin is paramagnetic, causing subtle changes in the magnetic resonance signal, which underlies the BOLD effect (Raimondo et al. 2021). Various analytical approaches can reveal distinct spatial and temporal brain patterns by examining specific frequency ranges in rs‐fMRI (Fasiello et al. 2022). Certain brain networks, such as the default and executive control networks, are delineated by their activity patterns across the entire brain. In contrast, local patterns are assessed using metrics like regional homogeneity (ReHo), amplitude of low‐frequency fluctuation (ALFF), and functional connectivity (FC) (Tolomeo and Yu 2022). ALFF/fALFF gauges the intensity of spontaneous BOLD activity in particular regions, whereas ReHo evaluates the similarity between the time series of a given voxel and its nearest neighbors. Both metrics demonstrate high reproducibility and possess clear physiological relevance.

FC between brain regions can provide insights into potential impairments in HIV patients. Previous rs‐fMRI studies have indicated increased ALFF in the right fusiform and left parahippocampal gyri, along with decreased ReHo in areas such as the anterior cingulate gyrus among individuals living with HIV (Ma et al. 2022). These alterations may be associated with visual and cognitive deficits that persist even after cART (Nguchu et al. 2021). However, there is a paucity of longitudinal studies, and evidence suggests ongoing cognitive decline following cART, possibly attributable to enduring neuroinflammation (Alagaratnam and Winston 2022). Previous research, often conducted on older patients with comorbidities, may lack robustness, underscoring the necessity for rigorously designed cognitive investigations in the context of HIV (Van den Hof et al. 2021).

In a prior cross‐sectional study, our team investigated the impact of HAND on FC, highlighting the vulnerability of visual networks and the significance of the middle frontal gyrus, vermis cerebellum, and insula (Han et al. 2022). Expanding on this work, we conducted a longitudinal study that integrated rs‐fMRI with neurocognitive assessments, explicitly focusing on the ANI stage. This investigation aimed to monitor cognitive fluctuations in ANI patients over time, particularly about distinct brain regions, and to examine potential correlations with clinical markers. Our hypothesis posits that early alterations in brain function in HIV are multifaceted, encompassing both cognitive deterioration and potential restoration in select brain areas.

## 2 Materials and Methods

### 2.1 Participants

This study received approval from the medical ethics committee at Beijing You'an Hospital, with all participants providing written informed consent by the Declaration of Helsinki. Between November 2020 and April 2023, 60 HIV‐infected patients with ANI were recruited, all of whom completed a one‐year follow‐up with an average interval of 1.68 years. In order to mitigate potential confounding variables, such as the known influences of gender and handedness on brain structure and function (Bao and Swaab 2011; Ristori et al. 2020; Tzourio‐Mazoyer et al. 2020), we established specific inclusion criteria. These criteria excluded right‐handed male participants, who comprise over 95% of our patient population. All participants were given standard antiretroviral therapy provided through the national free treatment program administered by the Chinese Center for Disease Control and Prevention (2021). The regimen consisted of tenofovir disoproxil fumarate, lamivudine, and efavirenz.

### 2.2 Inclusion and Exclusion Criteria

The inclusion criteria of this study included individuals who met the following requirements: (1) Chinese males, (2) aged between 20 and 50, and (3) right‐hand dominant. The exclusion criteria for participants included: (1) absence of brain tumors, infections, stroke, epilepsy, or other neurological diseases known to cause cognitive impairment; (2) absence of mental disorders caused by other medical conditions; (3) no history of illicit drug use or substance abuse/addiction; (4) no contraindications for MRI; and (5) participants had to be free from significant illnesses that could affect brain structure or function during the study. Additionally, all participants underwent assessments using the beck depression inventory (BDI) and the activities of daily living (ADL) scale to assess mood and daily functioning. Those with a BDI score exceeding 13, a history of major psychiatric disorders, or impaired ADL scores were excluded to prevent the inclusion of individuals at the MND stage or those with additional psychiatric comorbidities. HIV infection was verified through standard diagnostic protocols at certified medical institutions. Participants all underwent immunological Western blotting or polymerase chain reaction (PCR) analyses to confirm their HIV‐positive status. These diagnostic procedures were conducted as part of their regular clinical care before being enrolled in the study, guaranteeing precision in diagnosis with only individuals infected through homosexual intercourse being included in the study.

According to the Frascati criteria (Wei et al. 2020), the diagnostic classification of ANI necessitates fulfilling four conditions: (1) scoring at least one standard deviation below the mean of demographic‐adjusted normative scores in two or more cognitive domains; (2) maintaining stable daily life functioning; (3) not meeting criteria for delirium or dementia‐related impairments; and (4) showing no indication that other factors cause ANI. Before MR scanning, all participants underwent clinical evaluations and neurocognitive tests, with clinical data outlined in Table 1.

**TABLE 1:** Characteristics of participants at baseline and follow‐up.

Characteristics	Group	Mean	std	min	max
Age (years)	Baseline	35.03	8.16	20	50
	Follow‐up	36.70	7.84	21	51
Education (years)	Baseline/ follow‐up	14.20	3.27	9	20
Disease course (months)	Baseline	36.55	23.87	1	74
	Follow‐up	56.68	22.59	19	90
Nadir CD4	Baseline/ follow‐up	153.55	88.62	20.70	463.37
CD4+ T	Baseline	407.62	193.85	140.23	963.37
	Follow‐up	520.99	265.00	200.43	1100.12
CD4+/CD8+ ratio	Baseline	0.59	0.16	0.31	1.03
	Follow‐up	0.65	0.22	0.32	1.32
Abstraction/executive	Baseline	40.57	3.66	34	48
	Follow‐up	39.78	3.4	34	46
Memory (learning and recall)	Baseline	40.22	4.61	32	50
	Follow‐up	39.38	4.32	32	49
Attention/working memory	Baseline	42.11	3.55	36	49
	Follow‐up	41.00	3.14	36	49
Speed of information processing	Baseline	39.75	3.01	35	45
	Follow‐up	38.93	3.42	32	46
Verbal and language	Baseline	39.65	3.10	35	46
	Follow‐up	39.5	3.19	34	46
Fine motor skills	Baseline	39.77	3.05	34	45
	Follow‐up	39.68	3.07	34	46

Abbreviations: CD4 = cluster of differentiation 4; CD8 = cluster of differentiation 8; IQR = interquartile range; SD = standard deviation; TND = target not detected; T = T‐lymphocyte.

### 2.3 Neurocognitive Examination

This study conducted a thorough neurocognitive assessment covering six cognitive domains using established psychological test protocols (Liang et al. 2021). The domains included: (1) verbal and language abilities (assessed through the animal naming test); (2) attention and working memory (evaluated using the Wechsler Memory Scale‐III and the Paced Auditory Serial Addition Test); (3) memory function, encompassing learning and recall (measured by the Hopkins Verbal Learning Test‐Revised and the Brief Visuospatial Memory Test‐Revised); (4) speed of information processing (evaluated with the Trail‐Making Test Part A); (5) fine motor skills (assessed through the Grooved Pegboard Test); and (6) abstraction and executive function (evaluated using the Wisconsin Card Sorting Test 64‐card version) (Li et al. 2018). Neuropsychological performance was evaluated through the Frascati‐standardized battery, wherein raw scores were transformed into scaled scores and T‐scores. These T‐scores were normalized for age, gender, educational attainment, and the urban or rural background of the individuals, employing Chinese population‐specific normative data for culturally and clinically relevant comparisons. In cases where a cognitive domain comprised multiple tests, an average T‐score was calculated. Adjustments were made to test questions during follow‐up to reduce participant bias and interference (Tang et al. 2017).

### 2.4 MRI Data Acquisition

Imaging data were obtained using a 3.0T MR scanner (Tim‐Trio, Siemens, Erlangen, Germany) equipped with a 32‐channel head coil. Subjects were secured with tight foam pads to minimize motion artifacts during scanning. Participants were instructed to remain still, keep their eyes closed without falling asleep, relax, and refrain from engaging in specific thoughts while undergoing the MRI procedure. The rs‐fMRI data were acquired utilizing a gradient echo planar imaging sequence (Han et al. 2022). The acquisition parameters included: repetition time (TR), 2,000 ms; echo time (TE), 30 ms; flip angle, 90°; resolution matrix, 64 × 64; field of view (FOV), 224 × 224 mm; section thickness, 3.5 mm; the number of sections, 35; and voxel size = 3.5 × 3.5 × 3.5 mm^3^. In total, 35 axial slices and 240 time points were obtained for each subject. In addition, a sagittal magnetization prepared gradient‐echo (MPRAGE) sequence [repetition time (TR)/echo time (TE)/inversion time (TI) = 1,900/2.52/900 ms; acquisition matrix = 256 × 246; field of view = 250 × 250; flip angle = 9 degrees; voxel size = 1 mm × 0.977 mm × 0.977 mm] and axial T2‐weighted fluid‐attenuated inversion recovery (FLAIR) images (TR/TE/TI = 8000/2370.9/97; and matrix, 320 × 224) were also acquired for the exclusion of overt brain diseases such as stroke and demyelinating disease (Han et al. 2022).

### 2.5 Image Pre‐Processing

The rs‐fMRI data were pre‐processed using the data processing and analysis for brain imaging (DPABI V4.5) toolbox. The initial 10 time points were discarded to allow participants to acclimate to the scanning environment and stabilize the magnetic field. Subsequently, the remaining 230 time points underwent slice‐timing and realignment corrections to address head motion. Participants exhibiting translations exceeding 2.0 mm or rotations exceeding 2.0° during the MRI scan were excluded. Manual adjustments were made to the functional and structural images, including head rotation, translation, and origin positioning, as well as straightening of the entire image, by ensuring that Reorient Fun and Reorient T1 were checked. This manual adjustment process enhanced the accuracy of coregistration, segmentation, and normalization (Zhu et al. 2021). The rs‐fMRI data were then spatially normalized to the Montreal Neurological Institute (MNI) space and resampled to a voxel size of 3×3×3 mm3. Subsequently, to mitigate low‐frequency drift and high‐frequency physiological noise effects, all data underwent linear‐trend removal and temporal bandpass filtering (0.01 Hz < f < 0.08 Hz). The study implemented regression analysis to mitigate potential confounding effects, including head motion parameters, white matter, and cerebrospinal fluid signals (Meissner et al. 2020). The Friston 24‐parameter model was applied to address head motion impacts, while mean framewise displacement (FD) was included as a covariate in group analyses to further account for motion‐related residuals. Regarding the global signal regression, a review of relevant literature revealed conflicting findings, with some studies suggesting its removal could improve negative correlations or influence FC differences (Sobczak et al. 2021). After thoroughly examining this issue, the decision was made to proceed without global signal regression. The normalization process involved utilizing Diffeomorphic Anatomical Registration Through Exponentiated Lie Algebra to align structural and functional images, segmenting the structural image into gray matter, white matter, and cerebrospinal fluid, and creating transformation matrices to generate a group template that was ultimately standardized to the MNI space.

### 2.6 Computation of ALFF/fALFF Values and ReHo Maps

The ALFF for each voxel was determined by averaging the square root of the power spectrum within the frequency range of 0.01–0.08 Hz. To mitigate noise interference, the fractional ALFF (fALFF) was calculated as the ratio of ALFF to the total amplitude of low‐frequency oscillations (0‐0.25 Hz). As the complete amplitude spectrum needed to be computed, ALFF and fALFF were filtered in reverse order in the frequency domain. ReHo maps were generated by computing Kendall's coefficient of concordance (KCC) for each voxel and its 26 adjacent voxels (3×3×3‐1). For ReHo (0.01‐0.04 Hz), each subject's mean ReHo (mReHo) was derived by dividing each voxel's ReHo value by the entire brain's average ReHo value. Subsequently, all mReHo maps were smoothed using a Gaussian kernel with a full width at a maximum of 4 mm. Statistical analysis comparing baseline and follow‐up groups was conducted using paired t‐tests (p < 0.05), with results assessed through cluster‐level false discovery rate (FDR) correction requiring a minimum cluster size of 5 contiguous voxels. Alternatively, a voxel‐level threshold of p < 0.001 was applied for group comparison with a minimum cluster size of 10 contiguous voxels, without correction for multiple comparisons, to explore differences between groups.

### 2.7 Functional Connectivity

In the ROI‐based FC analysis, 16 brain regions exhibiting significant differences in ALFF/fALFF and Reho values between baseline and follow‐up periods, as determined by paired t‐tests, were designated regions of interest (ROIs). Utilizing DPABI software, the Pearson correlation coefficient (r) was computed between the time series of each ROI for each subject and the time series of all other ROIs to construct a comprehensive FC matrix comprising these ROIs. This process yielded a 16 × 16 FC correlation coefficient matrix comprising 16 × (16‐1)/2 r values. Subsequently, the FC matrix was transformed using Fisher Z for further analysis. To assess differences in FC between two groups across baseline and follow‐up periods, paired t‐tests were conducted using the GRETNA software platform to determine potential intergroup disparities in brain interval correlation coefficients within the FC matrix. Age, CD4+T cell count, CD4:CD8 ratio, and head movement parameters were included as covariates in the regression analysis. Significance was established at p< 0.05 following false discovery rate (FDR) multiple comparison correction, with a correction frequency of 120.

### 2.8 Statistical Analysis and Visualization

Paired t‐tests (satisfy normal distribution) and the Wilcoxon (non‐parametric tests) were used to analyze the group differences in demographics and clinical information. Multivariate linear regression was used to evaluate the neurocognitive test data (Matchanova et al. 2020). A significance threshold of p < 0.05 was set, and statistical analyses were conducted using SPSS software (version 26.0). Significant differences in ALFF/fALFF and ReHo between baseline and follow‐up groups were visualized using the DPABI software viewer and SPM12. The clustering of significant intergroup differences in ALFF/fALFF, ReHo, and FC was visualized using BrainNet Viewer and DPABI software viewers. ALFF/fALFF values, average ReHo values, and FC correlation coefficient values for these clusters were extracted using DPABI software. Pearson's correlation analysis was then applied to investigate the relationship between changes in ReHo/FC in ANI patients and clinical variables such as neurocognitive test scores and CD4+T cell counts.

## 3 Results

### 3.1 Participant Basic Information

**FIGURE 1:**
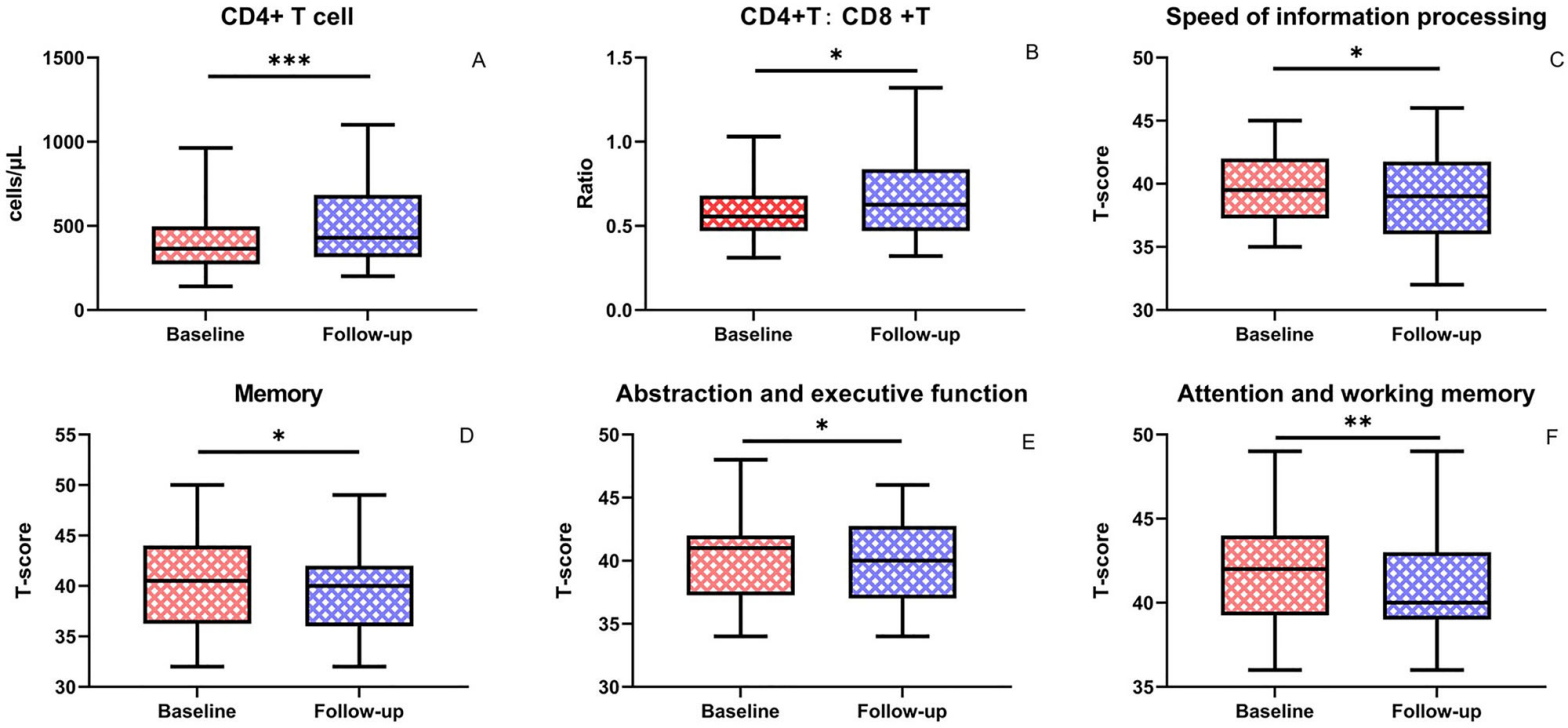
The differences of clinical characteristics and neurocognitive performance of the participants. Significance was set as p < 0.05. * p< 0.05; ** p< 0.01; *** p< 0.001.

The demographics, clinical characteristics, and neurocognitive test results at baseline and follow‐up are summarized in Table 1. Descriptive statistics show the mean and standard deviation for normally distributed variables and the median with the interquartile range (IQR) for non‐normally distributed variables. Inferential statistical comparisons between baseline and follow‐up measures are presented in Table 2. Statistically significant increases were observed in CD4+ T‐cell counts (p = 0.009). At the same time, reductions were noted in T‐scores for memory (p = 0.021), speed of information processing (p = 0.023), abstraction/executive function (p = 0.032), and attention/working memory (p = 0.011). No significant change was found in the CD4+/CD8+ ratio (p = 0.056) (Figure 1).

**TABLE 2:** Inferential statistical analyses of characteristics.

Characteristics	Baseline	Follow‐up	p value
Age(years)	35.03 (20‐50)	36.70 (21‐51)	0.000^a^
Disease course(months)	36.55 (1‐74)	56.68 (19‐90)	0.000^a^
CD4+ T	407.62(140.23‐963.37)	520.99 (200.43‐1100.12)	0.000^ a^
CD4+/CD8+ ratio	0.59 (0.31‐1.03)	0.65 (0.32‐1.32)	0.035^ a^
Speed of information processing	39.75 (35‐45)	38.93 (32‐46)	0.023 ^a^
Memory (learning and recall)	40.22±4.61	39.38±4.32	0.021 ^a^
Abstraction/executive	40.57 (34‐48)	39.78 (34‐46)	0.032 ^a^
Attention/working memory	42.12 (36‐49)	41.00 (36‐49)	0.009 ^a^
Verbal and language	39.65 (35‐46)	39.5 (34‐45)	0.054 ^a^
Fine motor skills	39.77 (34‐45)	39.68 (34‐46)	0.068 ^a^

*Note*: The clinical data and neurocognitive examination scores between the two groups were compared by a paired t‐test or Wilcoxon test test. Significance was set as p < 0.05. ^a^ paired t‐test; ^b^ Wilcoxon test Test;

Abbreviations: SD‐standard deviation;CD4‐ cluster of differentiation 4; CD8‐ cluster of differentiation 8; T‐ T‐lymphocyte.

### 3.2 Differences in ALFF/fALFF

**FIGURE 2:**
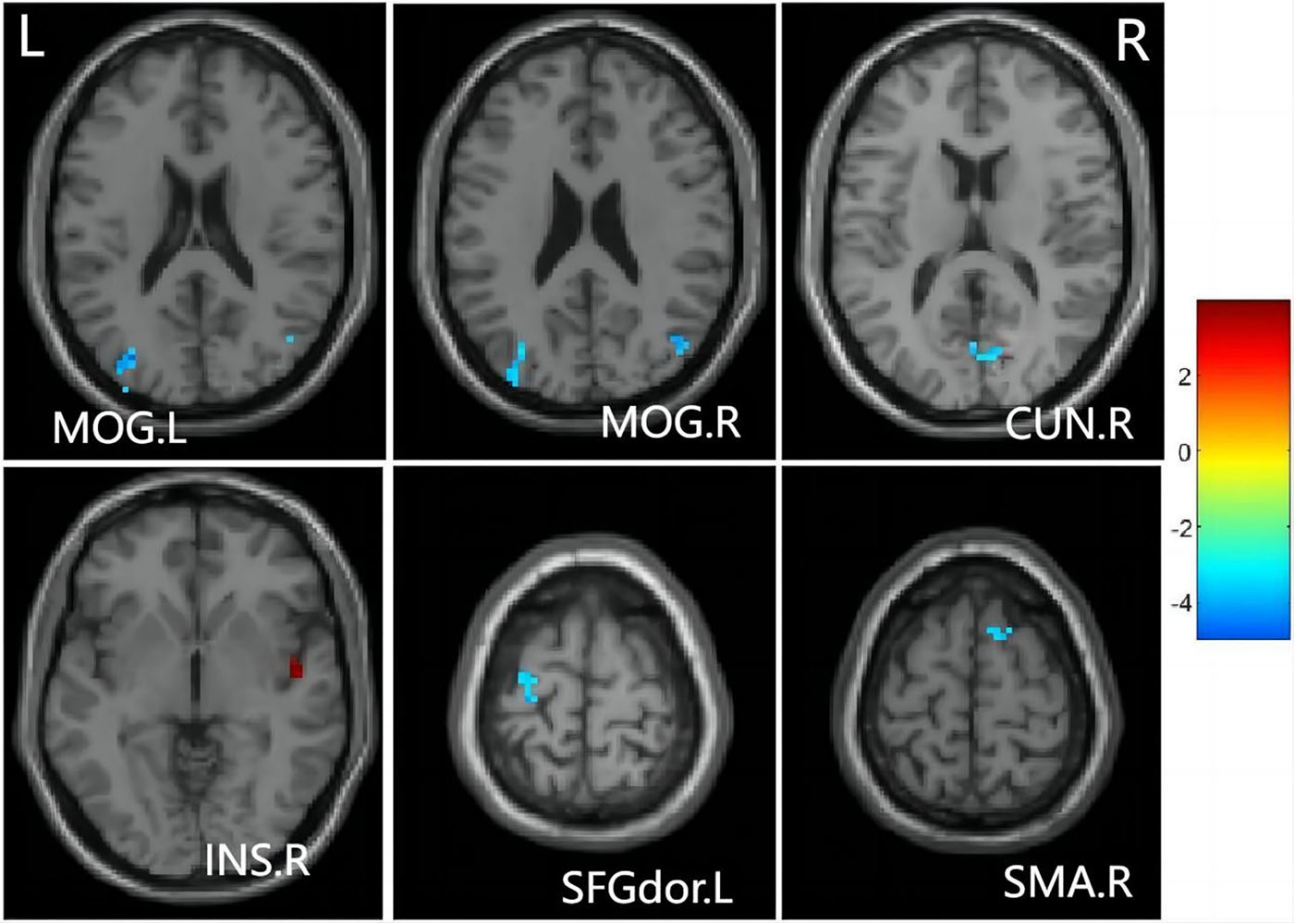
Brain regions with different ALFF. MOG.L, left middle occipital gyrus; MOG.R, right middle occipital gyrus; CUN.R, right cuneus; SFGdor.L, left superior frontal gyrus, dorsolateral; SMA.R, right supplementary motor area; INS.R, right insula; The color bar indicates a scale of T values.

**FIGURE 3:**
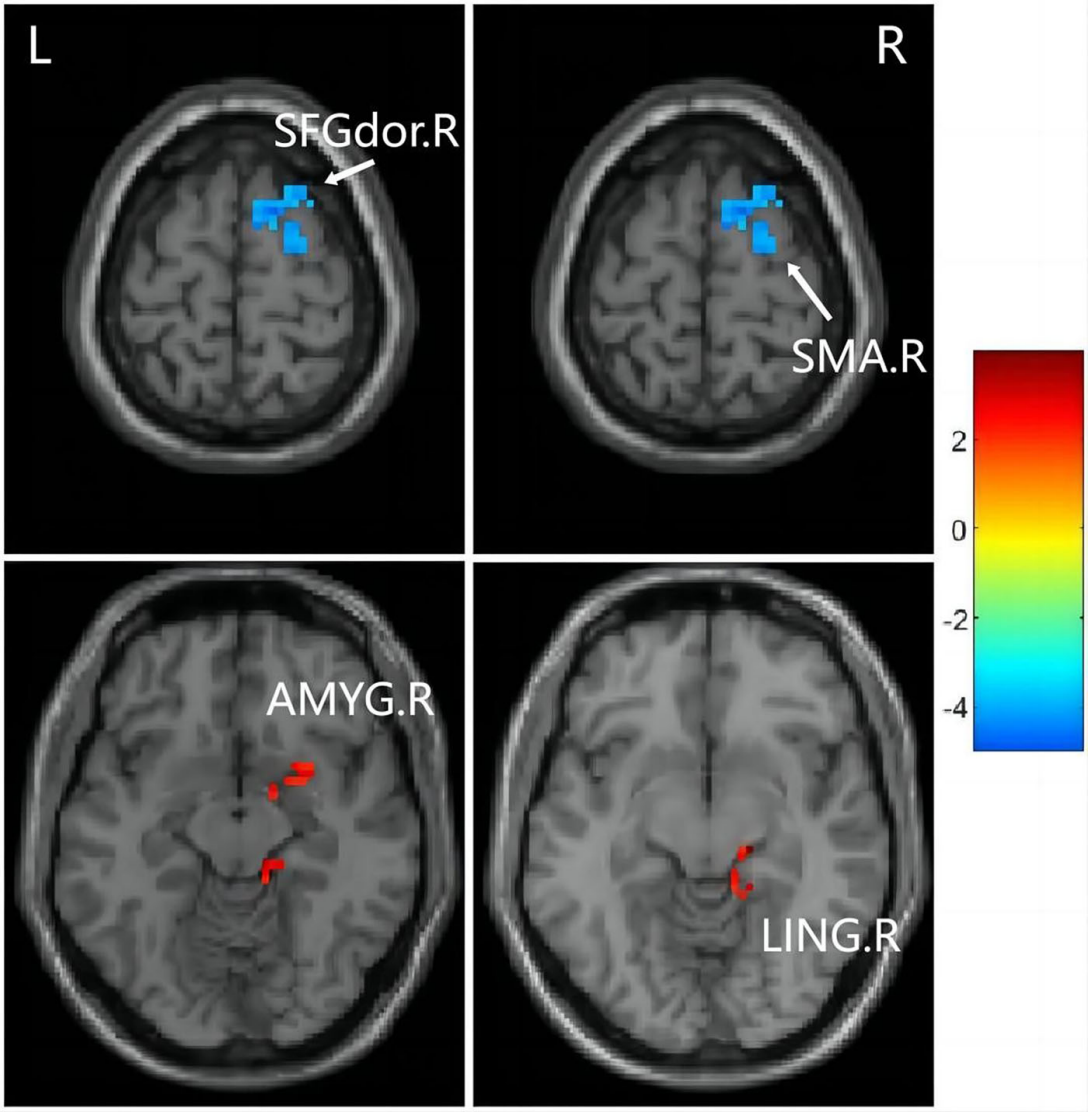
Brain regions with different fALFF. LING.R, right lingual gyrus; AMYG.R, right amygdala; SFGdor.R, right superior frontal gyrus, dorsolateral; SMA.R, right supplementary motor area; The color bar indicates a scale of T values.

A paired t‐test revealed significant group differences in ALFF and fALFF between the baseline and follow‐up groups. No significant group differences in ALFF were observed using a stringent threshold (cluster‐level p < 0.05 with False Discovery Rate correction). However, employing a less stringent threshold (cluster‐level uncorrected p < 0.001, cluster size > 10), the follow‐up group exhibited a notable ALFF decrease in the left middle occipital gyrus (MOG.L), right middle occipital gyrus (MOG.R), right cuneus (CUN.R), left dorsolateral superior frontal gyrus (SFGdor.L), and right supplementary motor area (SMA.R), along with ALFF enhancement in the right insula (INS.R) when compared to the baseline group (Figure 2 and Table 3). Similarly, no significant group differences in fALFF were identified using a strict threshold (cluster‐level p < 0.05 with False Discovery Rate correction). Nevertheless, utilizing a less strict threshold (cluster‐level uncorrected p < 0.001, cluster size > 10), the follow‐up group displayed a significant fALFF decrease in the right superior frontal gyrus (SFGdor.R) and SMA.R, as well as fALFF enhancement in the right lingual gyrus (LING.R) and right amygdala (AMYG.R) compared to the baseline group (Figure 3 and Table 4).

**TABLE 3:** Peak MNI coordinates (X, Y, Z) of brain regions showing significant ALFF value changes.

Brain regions	Peak MNI, mm			T‐score	Size	p‐value
	X	Y	Z			
Left middle occipital gyrus	30	−78	18	−3.99	14	0.0004
Right middle occipital gyrus	45	−69	24	−4.08	15	0.0002
Right cuneus	6	−78	15	−3.55	10	0.0009
Left superior frontal gyrus, dorsolateral	−27	−9	69	−3.65	11	0.0008
Right supplementary motor area	9	12	69	−3.82	13	0.0006
Right insula	48	−6	−3	3.93	14	0.0004

*Note*: A positive t value represents increased ALFF, whereas a negative t value represents decreased ALFF. The unit of size is voxel. The significance threshold was set at p < 0.001, cluster ≥ 10.

Abbreviation: MNI‐ Montreal Neurological Institute.

**TABLE 4:** Peak MNI coordinates (X, Y, Z) of brain regions showing significant fALFF value changes.

Brain regions	Peak MNI, mm			T‐score	Size	p‐value
	X	Y	Z			
Right supplementary motor area	24	15	66	−3.96	14	0.0004
Right superior frontal gyrus, dorsolateral	21	−3	66	−3.53	10	0.0009
Right amygdala	15	0	−15	5.05	25	0.0001
Right lingual gyrus	18	−24	−9	4.79	20	0.0001

*Note*: A positive t value represents increased fALFF, whereas a negative t value represents decreased fALFF. The unit of size is voxel. The significance threshold was set at p < 0.001, cluster ≥ 10.

Abbreviation: MNI‐ Montreal Neurological Institute.

### 3.3 Differences in ReHo

**FIGURE 4:**
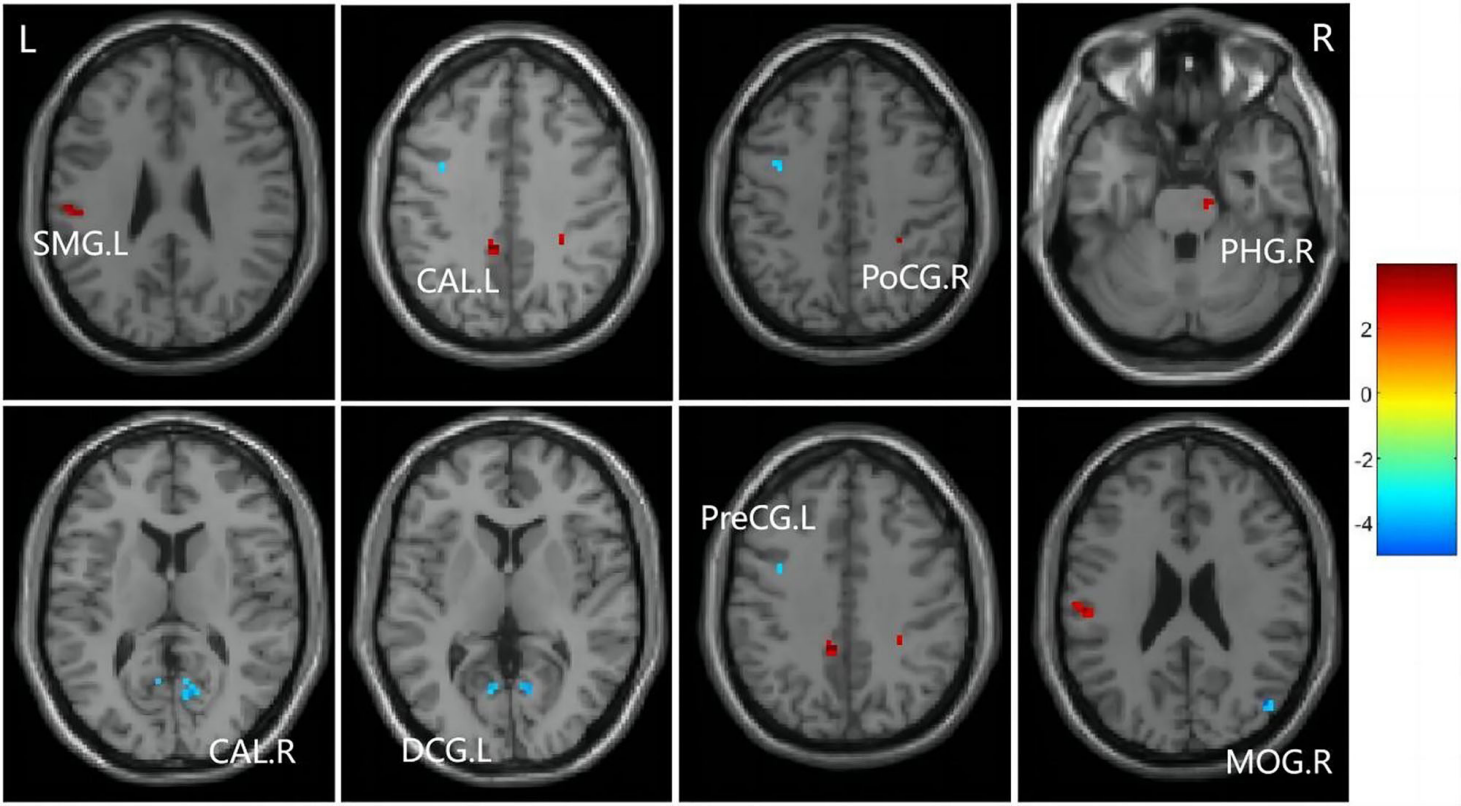
Brain regions with different ReHo maps. SMG.L, left supramarginal gyrus; DCG.L, left median cingulate and paracingulate gyri; PoCG.R, right postcentral gyrus; PHG.R right parahippocampal gyrus; CAL.R, right calcarine fissure and surrounding cortex; CAL.L, left calcarine fissure and surrounding cortex; MOG.R, right middle occipital gyrus; PreCG.L, left precental gyrus; The color bar indicates a scale of T values.

The statistical methods previously outlined were applied, with ReHo demonstrating notably more robust outcomes. In comparison to the baseline group, the follow‐up group exhibited a significant decrease in ReHo in specific brain regions, including the left median cingulate and paracingulate gyri (DCG.L), right calcarine fissure and surrounding cortex (CAL.R), MOG.R, and left precentral gyrus (PreCG.L). Conversely, ReHo was enhanced in the left supramarginal gyrus (SMG.L), right postcentral gyrus (PoCG.R), right parahippocampal gyrus (PHG.R), and left calcarine fissure and surrounding cortex (CAL.L) (FWE corrected, voxel‐level P < 0.001, cluster‐level P < 0.05), as illustrated in Figure 4 and Table 5.

**TABLE 5:** Peak MNI coordinates (X, Y, Z) of brain regions showing significant ReHo value changes.

Brain regions	Peak MNI, mm			T‐score	Size	p‐value
	X	Y	Z			
Left median cingulate and paracingulate gyri	9	−60	9	−5.72	5	0.03
Right calcarine fissure and surrounding cortex	12	−63	9	−5.81	6	0.03
Right middle occipital gyrus	48	−72	24	−6.09	7	0.02
Left precental gyrus	−39	3	42	−5.61	5	0.03
Left supramarginal gyrus	−54	−21	24	7.24	12	0.01
Left calcarine fissure and surrounding cortex	30	−39	39	6.70	10	0.01
Right postcentral gyrus	−6	−42	39	7.90	16	0.01
Right parahippocampal gyrus	12	−21	−24	5.57	5	0.03

*Note*: A positive t value represents increased ReHo, whereas a negative t value represents decreased ReHo. The unit of size is voxel. The significance threshold was set at p< 0.05(After FDR correction).

Abbreviation: MNI‐ Montreal Neurological Institute.

Difference in ROI‐based FC and correlations of the imaging alterations with clinical variables and neurocognitive examination.

**FIGURE 5:**
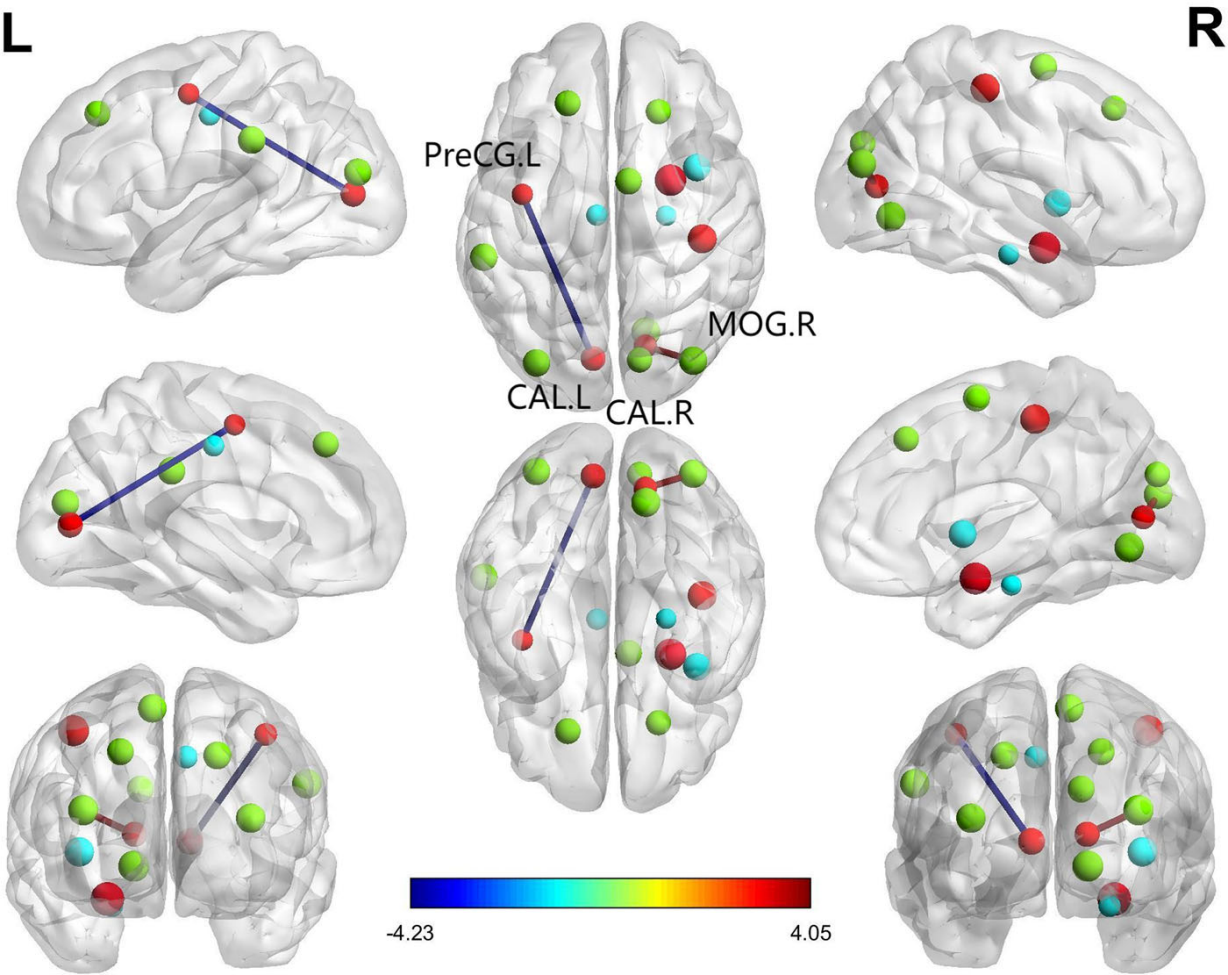
Differences in ROI region functional connectivity between the two groups. Compared with the baseline group, patients showed decreased FC between the CAL during the follow‐up group. L and PreCG. L, while the FC between the right prefrontal cortex CAL. R and the MOG. R increased. The results were corrected for multiple comparisons (FDR correction, p<0.05). The line represents the FC, with significant differences between each pair of defined ROI regions in the two groups. Red represents a positive t‐value indicating an increase in FC during the follow‐up period compared to baseline, while blue represents a negative t‐value indicating a decrease in FC during the follow‐up period.

**FIGURE 6:**
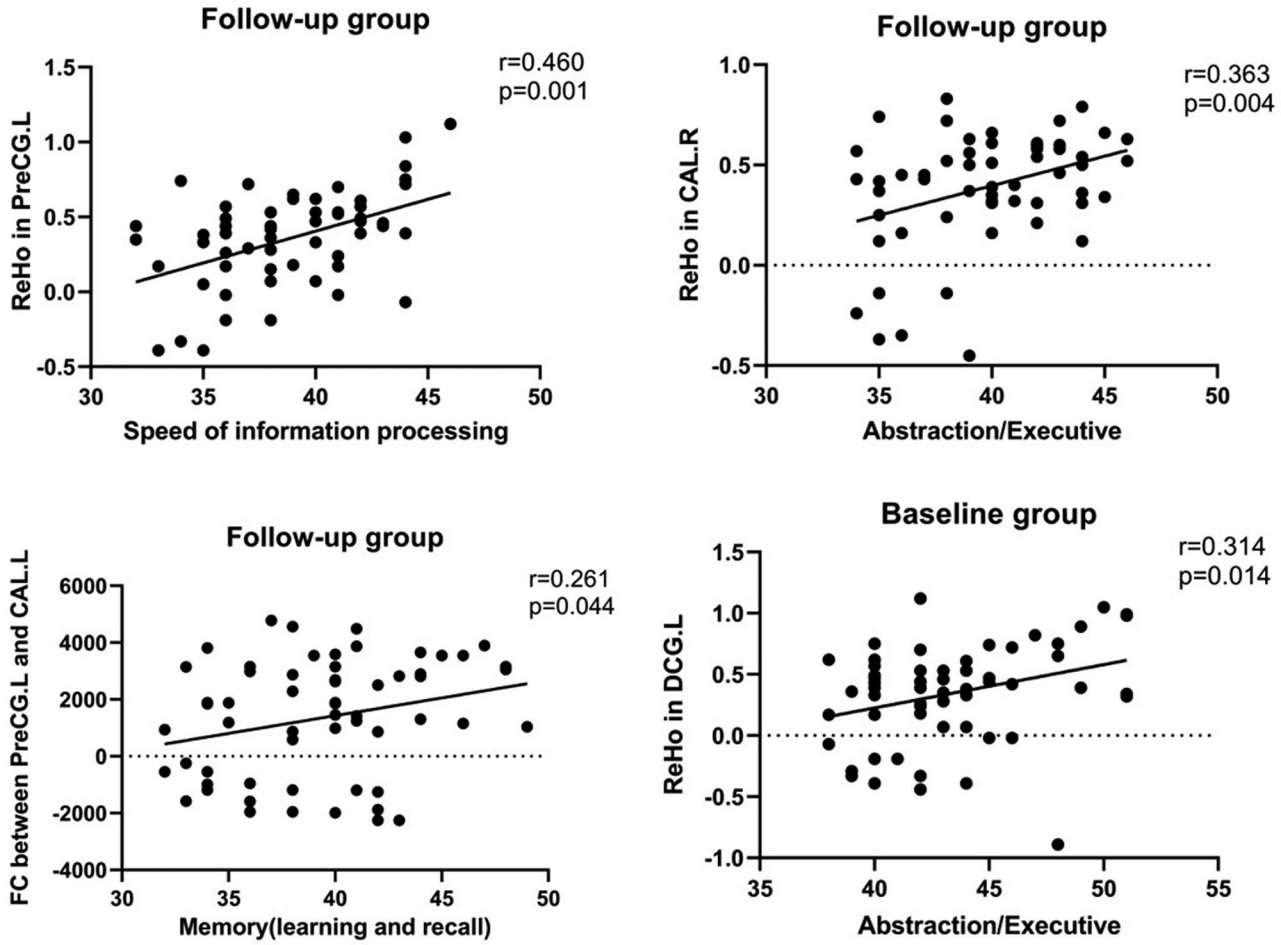
Correlation between abnormal ReHo/FC and clinical variables in baseline group with baseline and follow‐up group. The threshold was set at p < 0.05 without multiple comparison corrections.

A paired t‐test indicated significant variations in FC between the baseline and follow‐up cohorts. In Figure 5, the findings demonstrate decreased FC among individuals with ANI in the follow‐up group. Specifically, reductions were observed between the left precentral gyrus (PreCG.L) and left calcarine sulcus (CAL.L), as well as between the MOG.R and CAL.R, compared to the baseline group. Higher mReHo values in the PreCG.L within the follow‐up group positively correlated with processing speed (r = 0.460, p = 0.001), whereas elevated ReHo values in the CAL.R were linked to abstraction and executive function (r = 0.363, p = 0.004), as illustrated in Figure 6. Moreover, a notable increase in FC z‐scores between PreCG.L and CAL.L in the follow‐up group was positively correlated with memory performance (r = 0.261, p = 0.044). Conversely, in the baseline group, elevated ReHo values in the left dorsal cingulate gyrus (DCG.L) were positively correlated with abstraction and executive function (r = 0.314, p = 0.014). Notably, all reported outcomes underwent adjustment for multiple comparisons (false discovery rate < 0.05).

## 4 Discussion

This study utilizes ALFF/fALFF, ReHo, and ROI‐based FC analyses to examine alterations in brain regions and functional connectivity in ANI patients pre‐ and post‐follow‐up. While individual‐level changes may not be clinically significant, our study focuses on group‐level changes that can sensitively capture subtle yet meaningful shifts in intrinsic brain activity. Despite cART treatment, ANI patients exhibit ongoing functional network modifications linked to specific clinical characteristics and neurocognitive functions. Notably, all participants underwent screening to eliminate confounding factors such as current substance use, ensuring that observed neural changes are more likely associated with HIV pathophysiology and treatment effects rather than external influences. These findings offer valuable insights into the mechanisms driving functional network disruptions in individuals with HIV infection.

Our study found a decrease in mReHo values in several brain regions, including DCG.L, CAL.R, MOG.R, and PreCG.L, in the follow‐up group compared to baseline. Notably, the middle occipital gyrus (MOG) showed reductions in ALFF and ReHo values, indicating its potential importance in the observed functional changes in patients with ANI. The primary visual cortex in the occipital lobe is crucial for processing visual information such as shapes, colors, and movements (Montgomery et al. 2022). Visual stimuli travel from the retina to the primary visual cortex through the geniculate nucleus of the lateral thalamus and the optic tract. The MOG contributes to spatial perception and the integration of visual information for stereo vision (Li et al. 2024). Previous studies have linked abnormalities in occipital lobe function to cognitive deficits in individuals with HIV (Wiesman et al. 2018). For example, Wiesman showed that atypical dynamics in the occipital lobe could distinguish cognitive impairment in HIV patients. Additionally, Liu's research suggested an inverse relationship between visual cortex function and declines in the posterior cingulate cortex and left angular gyrus of the default mode network (DMN) in HIV patients, while the occipital lobe volume positively correlated with visual‐related cognitive function (Liu et al. 2020). Our results are consistent with these findings, as we observed a positive correlation between the strength of functional connections linking MOG.R and CAL.R and information processing speed in cognitive assessments. This implies that declines in visual‐related cognitive functions could predict disease progression in ANI patients.

The decline in ReHo values across various brain regions highlights their diverse functional importance. The median cingulate and paracingulate gyri (DCG) are pivotal in receiving inputs from the amygdala, orbitofrontal gyrus, and medial frontal gyrus and in transmitting signals to the anterior cingulate gyrus and striatum, crucial for executive control, emotional processing, and pain perception (Ma et al. 2023). Damage to these regions has been linked to altered decision‐making in HIV patients, leading to a preference for ambiguous choices over high‐risk decisions (Hall et al. 2021). This aligns with the observed decreased information processing speed in the follow‐up group. Furthermore, the bilateral calcarine fissure and surrounding cortex, essential for visual information processing in the visual cortex, showed reduced ReHo and ALFF values. Specifically, changes in ReHo values pre‐ and post‐follow‐up indicated increased activation on the left and decreased activation on the right, suggesting hemispheric asymmetry. This asymmetry was positively associated with abstraction and executive function scores, indicating a connection between modifications in visual‐related brain regions and cognitive performance (Wang et al. 2023). Moreover, the precentral gyrus (PreCG), crucial for motor function regulation and proprioception, displayed impaired function (Silva et al. 2022). A study by Li J et al. (2021) on HIV‐infected adolescents revealed a correlation between decreased PreCG volume and reduced motor capacity. These results suggest that alterations in visual cortex function significantly affect information processing speed and executive functions, while motor impairments in HIV patients may become evident at a later stage.

Meanwhile, ReHo values were increased in the SMG.L, PoCG.R, and PHG.R brain regions, which are known for their involvement in processing auditory, somatosensory, cognitive, emotional control, and visual information (Xiang et al. 2023). This phenomenon extends beyond HIV‐infected individuals, as evidenced by a longitudinal study on patients receiving continuous cART treatment, which showed heightened functional connectivity in the postcentral gyrus and hippocampal gyrus, as well as increased gray and white matter volume one to two years post‐acute phase (Haynes et al. 2018). The elevated ReHo values suggest enhanced synchronization among neurons and neighboring tissues, potentially linked to immune reconstitution following antiretroviral therapy (Lai et al. 2022). The positive correlation between ReHo values and memory functions implies that enhanced synchronization in these brain regions may be associated with cognitive changes. However, despite this, memory‐related functions in HIV patients exhibit a progressive decline over time, suggesting that this alteration may represent a compensatory mechanism, highlighting the complex nature of brain function adaptations in HIV patients.

The ALFF and fALFF results, although not entirely statistically robust, exhibit good stability and repeatability, rendering them valuable for our study. fALFF offers greater specificity for gray matter signals but lower repeatability than ALFF. Therefore, combining ALFF and fALFF can enhance the analysis. Decreased ALFF and fALFF values are linked to brain regions implicated in visual information processing, self‐regulated movement, executive functions, and emotional control (Lenroot and Giedd 2006). In conjunction with the amygdala, the insula and prefrontal cortex are pivotal for somatosensory and motor functions, emotional regulation, and homeostasis maintenance. PET‐CT studies have revealed a significant reduction in serotonin transporter binding in the insula of HIV patients, potentially contributing to elevated rates of depressive symptoms (Hammoud et al. 2010).

In rs‐fMRI analysis, functional segregation pertains to examining individual brain regions, with the ALFF being a suitable metric for this purpose (Zhang et al. 2021). On the other hand, functional integration involves studying interactions between brain regions, commonly assessed through methods such as seed‐based and ROI‐based FC. ReHo combines aspects of both segregation and integration by measuring the local coherence among neighboring voxels (Zhao et al. 2022). Although ReHo alone may not comprehensively explain the local neural activity, its integration with other rs‐fMRI metrics can enhance the overall interpretative capacity (Li et al. 2021; Yang et al. 2021). The robust ReHo findings observed in our study underscore the dynamic changes in brain function among individuals with ANI during follow‐up, emphasizing the importance of ReHo in elucidating alterations in functional brain networks.

Our cohort demonstrated a low mean CD4/CD8 ratio, consistent with previous findings indicating that a considerable number of individuals living with HIV exhibit ratios below the conventional threshold of 1.0, even when undergoing antiretroviral therapy (Serrano‐Villar et al. 2014). This population's median CD4/CD8 ratio typically falls between 0.55 and 0.75, highlighting persistent immune imbalance. However, all participants in our study were virally suppressed and free from intracranial opportunistic infections or other confounding conditions that could affect neuroimaging or neurocognitive results. Therefore, although the CD4/CD8 ratio reflects ongoing immune challenges, it may not compromise the internal validity of our findings.

Furthermore, while cART has significantly reduced the incidence of severe HAND, its impact on milder forms such as ANI remains complex. Several studies have raised concerns regarding the potential neurotoxicity of certain antiretroviral agents, particularly those with high central nervous system (CNS) penetration‐effectiveness scores (Lanman et al. 2021; Lin et al. 2021). Long‐term cART exposure has been implicated in mitochondrial dysfunction, oxidative stress, and neuronal injury, potentially exacerbating subtle cognitive impairments in virally suppressed individuals (Gonzalez et al. 2021; Yuan and Kaul 2021). However, the evidence remains inconclusive, and the neuroprotective versus neurotoxic effects of cART may vary depending on individual drug regimens, patient characteristics, and duration of treatment. Our findings should thus be interpreted within the context of ongoing debates on cART's dual role, underscoring the need for further longitudinal studies incorporating detailed ART regimen analyses and CNS biomarker data.

This study has several limitations. Firstly, the study did not consider the influence of antiretroviral use as a classification condition within or between groups, which could have affected the results. The participants' varying drug usage may have influenced the outcomes, as highlighted in previous studies (Wallet et al. 2019; Nicol and McRae 2021). Secondly, this study was limited by the lack of inclusion of additional biomarkers such as interleukin‐6 (IL‐6), C‐reactive protein (CRP), and D‐dimer (Boulware et al. 2011; Nixon and Landay, 2010), which play crucial roles in characterizing systemic inflammation and coagulopathy associated with HIV. Despite the analysis of CD4+ T‐cell count, the absence of these biomarkers hinders a thorough assessment of the biological complexity and inflammatory burden of HIV in this cohort. Thirdly, although participants were screened using the BDI and ADL assessments to rule out major depressive symptoms and functional impairments, we did not examine the relationship between neuroimaging findings and broader mental health metrics beyond cognitive domains. This lack of in‐depth psychiatric profiling represents a limitation of the present study and should be addressed in future research. Fourthly, the subjective selection of seed points may introduce bias, and the lack of a healthy control group hinders the ability to determine whether the observed brain changes are abnormal. Lastly, transmission occurs mainly among men who have sex with men in this region, the absence of female participants constrains the applicability of the results to the broader population. Future research endeavors should involve the integration of graph theory or independent component analysis to strengthen the robustness of these findings.

## 5 Conclusion

This study uncovers notable alterations in brain function among HIV patients with ANI receiving ongoing cART. The study reveals reductions in ALFF and fALFF in critical brain regions involved in visual processing and motor function and decreased ReHo in areas essential for cognitive control. Conversely, increased ReHo in other regions indicates a potential compensatory mechanism. The observed changes in FC were associated with cognitive deterioration, underscoring the necessity for continuous neuroimaging to monitor these changes. These results underscore the significance of incorporating various rs‐fMRI metrics to enhance the comprehension and management of HIV‐related neurocognitive disorders.

## Author Contributions

**Juming Ma**: conceptualization, data curation, formal analysis, methodology, project administration, supervision, writing–review and editing. **Zhongkai Zhou**: conceptualization, methodology, supervision. **Shuai Han**: data curation, investigation. **Chuanke Hou**: conceptualization, validation. **Xingyuan Jiang**: writing–original draft, validation. **Fan Xu**: data curation, validation. **Haixia Luo**: conceptualization. **Jiaojiao Liu**: software, visualization. **Wei Wang**: investigation, visualization. **Haiyan Zhao**: formal analysis, software. **Lingling Zhao**: conceptualization, data curation, methodology, writing–review and editing, resources. **Hongjun Li**: resources, conceptualization, data curation, funding acquisition, project administration, supervision, writing–review and editing, writing–original draft.

## Ethics Statement

The research involving human subjects underwent review and approval by the Beijing You'an Hospital Ethics Committee. All patients/participants gave written informed consent to partake in the study, including consent to publish potentially identifiable images or data in this article.

## Peer Review

The peer review history for this article is available at https://publons.com/publon/10.1002/brb3.70559

## Data Availability

The authors confirmed that the data supporting the findings of this study are present within the manuscript.
